# Learning About the Current State of Digital Mental Health Interventions for Canadian Youth to Inform Future Decision-Making: Mixed Methods Study

**DOI:** 10.2196/30491

**Published:** 2021-10-19

**Authors:** Jessica Kemp, Jill Chorney, Iman Kassam, Julie MacDonald, Tara MacDonald, Lori Wozney, Gillian Strudwick

**Affiliations:** 1 Campbell Family Mental Health Research Institute Centre for Addiction and Mental Health Toronto, ON Canada; 2 Institute of Health Policy, Management and Evaluation University of Toronto Toronto, ON Canada; 3 Mental Health and Addictions IWK Health Halifax, NS Canada; 4 Department of Psychiatry Dalhousie University Halifax, NS Canada; 5 Mental Health and Addictions Nova Scotia Health Sydney, NS Canada; 6 Mental Health and Addictions Nova Scotia Health Port Hawkesbury, NS Canada; 7 Mental Health and Addictions Policy and Planning Nova Scotia Health Dartmouth, NS Canada

**Keywords:** youth mental health, digital mental health, COVID-19, digital mental health interventions, e-mental health

## Abstract

**Background:**

The COVID-19 pandemic has increased the demand for youth mental health services in Canada as disruptions to clinical care continue to persist due to the risk of transmission and exposure to the virus. Digital mental health interventions, including web-based resources and mobile apps, have provided opportunities to support youth mental health remotely across Canada. There is a need to better understand how these digital interventions are being selected, recommended, and used in various regions across Canada.

**Objective:**

A national jurisdictional scan was completed to (1) determine what web-based programs, apps, and websites are promoted and licensed in Canada for youth mental health; (2) identify criteria and decision-making processes that Canadian jurisdictions use to select web-based programs, apps, and websites for youth mental health; and (3) identify upcoming trends, innovations, and digital mental health possibilities that are emerging in the youth sector.

**Methods:**

The aims of the jurisdictional scan were addressed through a review of related academic and grey literature; stakeholder interviews, including individuals involved in various areas of the youth mental health sector; and a social media review of pertinent Twitter content.

**Results:**

A total of 66 web-based resources and apps were identified for use by youth in Canada. 16 stakeholder interviews were completed and included discussions with researchers, clinicians, youth organizations, and others involved in digital interventions for youth mental health. These discussions identified a limited use of frameworks used to guide decision-making processes when selecting digital interventions. Many clinicians agreed on a similar set of eligibility requirements for youth mental health apps and digital resources, such as the evidence base and cultural relevance of the intervention. Stakeholders also identified upcoming trends and innovations in the youth digital mental health space, including artificial intelligence, digital phenotyping, and personalized therapy. Over 4 weeks, 2184 tweets were reviewed to identify and compare global and national trends and innovations involving digital mental health and youth. Key trends included the promotion of regional chat services as well as the effects of the COVID-19 pandemic on youth mental health and access to care.

**Conclusions:**

As organizations begin to plan for the delivery of mental health care following the pandemic, there are concerns about the sustainability of these digital mental health interventions as well as a need for services to be more informed by the experiences and preferences of youth.

## Introduction

Canadian jurisdictions have significantly altered their delivery of mental health care for youth over the past year, given the rising need for mental health services related to the COVID-19 pandemic [1]. A Canadian study indicated that 67% to 70% of children and adolescents aged 6-18 years have experienced a deterioration in one or more mental health domains because of changes such as stress and social isolation during the pandemic [1]. Organizations have broadened their use of virtual care and digital mental health interventions, including web-based programs, apps, and websites [2]. In consideration of the rapid shift to the use of digital mental health services and interventions, this review can support Canadian decision-makers and leaders in understanding these changes and how youth mental health is supported through digital interventions. To accomplish this, a jurisdictional scan was completed to address current needs and future directions in the youth mental health space.

To identify digital mental health resources, criteria, and decision-making processes used to select resources, and upcoming trends and innovations, the following aims were established:

1. Determine what web-based programs, apps, and websites are promoted and/or licensed by Canadian jurisdictions (eg, health authorities, national youth mental health organizations, provinces, and territories) for youth mental health.
2. Identify criteria and decision-making processes that Canadian jurisdictions use to select web-based programs, apps, and websites for youth mental health.
3. Identify upcoming trends, innovations, and digital mental health possibilities that are emerging in the youth sector.

## Methods

A review of current academic and grey literature was completed, including searches of databases (eg, PubMed, University of Toronto Libraries), reports, working papers, and presentations from Canadian digital health organizations. Titles and abstracts were reviewed to determine if information about youth mental health and digital interventions was included. Sources that met this inclusion criterion were then subjected to rapid content analysis and were organized based on identified themes. App stores, including the Google Play Store and Apple App Store, were also reviewed to identify digital mental health apps currently available to youth in Canada.

A series of key stakeholder interviews were conducted with representatives across Canada. Single interviews were conducted by two members of the research team via videoconferencing or telephone and followed a semistructured design based on a set of six questions. Participants were asked to discuss the criteria their organization uses to select resources for youth or decision-making processes/appraisals that are used to evaluate digital re**s**our**c**es such as websites and mobile apps. Trending and emerging innovations were also discussed, and all participants were able to provide additional information relevant to the topic. One research member took notes during each interview, and this information was later used for thematic analysis to identify key themes.

Data mining techniques and content analysis were used to retrieve and analyze information collected from the Twitter application programming interface over 4 weeks using a Twitter Archiving Google Sheet (TAGS). As Twitter is a platform commonly used by researchers, clinicians, youth organizations, health care leaders, and others involved in youth mental health care, Twitter data provided a snapshot of in-the-moment insights into trending topics and innovations related to digital mental health interventions for youth during the COVID-19 pandemic. These trends were identified to understand the similarities or disparities between the experiences of national and international stakeholders.

Further information about the methodology used for this research is available upon request from the corresponding author.

## Results

### Review of Web-Based Resources, Apps, and Related Literature

A total of 66 digital interventions were identified as promoted or licensed by Canadian jurisdictions and organized based on their delivery methods, including web-based resources or mobile apps. Information was recorded for each resource based on the American Psychiatric Associations App Evaluation Model [3]. A complete list of the digital interventions, including web-based resources, and associated criteria and tags are available in Multimedia Appendix 1, and the mobile apps are provided in Multimedia Appendix 2.

The review of related literature identified current trends in the youth digital mental health space. A common theme that was highlighted throughout the review of the literature was the limited availability of evidence-based apps. Numerous reviews revealed that formal evaluation methods were often not used to support the use of mobile apps for youth mental health, raising concerns about the efficacy of digital interventions used in clinical settings [4-8]. Researchers recommended that more rigorous testing be done to provide high-quality evidence for the use of digital interventions for youth mental health [8].

Research studies also reviewed the use of evaluation methods for digital mental health interventions for youth and provided various metrics used to quantify effectiveness and improvements to mental health [9-11]. A systematic review included metrics such as mental health outcome data, health service outcome data, technology use, and feedback from children and youth [9]. Other evaluation methods included clinical outcomes of randomized control trials to determine the effectiveness of digital mental health interventions [10].

Co-design was identified as a powerful tool in the development of digital mental health interventions for children and youth. Researchers indicated that there is a need for co-design with youth, including “creative collaboration, varying stages of involvement, collaboration activities, user-engagement techniques, prototype development, and consideration for diversity within youth populations” [12].

Most of the articles focused on the development and use of mobile apps targeting child and youth experiences with anxiety and depression [13-16]. There was a paucity of research on other types of digital mental health interventions as well as any digital interventions for eating disorders and many complex mental illnesses, with only one article identified on youth experiences with psychosis [17].

Gamification was also identified as a prominent topic in the results of the review. Some considerations were discussed that can address the needs of specific youth groups who may benefit from the use of personalized game-based interventions [18-21]. Some considerations include the integration of video games with clinical care processes and programming, the use of gender-diverse characters in game-based digital mental health interventions, and a focus on the prevention of mental illness as opposed to treatment-based games [18].

### Stakeholder Interviews

A total of 16 stakeholders, including clinicians, researchers, youth organizations, online therapists, business analysts, and others involved in the youth mental health space, took part in the interviews. Organizations included representation from numerous regions within Canada, including Ontario, British Columbia, Alberta, and Nova Scotia, as well as the US state of Massachusetts. Stakeholders discussed the digital mental health interventions they currently use, that their organization endorses, or that they are involved in for research purposes.

Additionally, the stakeholders discussed the need for improved guidance when selecting and appraising digital mental health interventions for youth. Overall, most respondents relied on individual selection criteria to inform their decision-making processes as opposed to standardized frameworks. Table 1 includes a list of specific appraisal tools referenced by participants as well as individual criteria cited by respondents as informing their decision-making processes.

**Table 1:** Decision-making processes and appraisal methods used for selecting digital mental health interventions for youth identified during stakeholder interviews.

Process or appraisal method		The rationale for process or appraisal
**Frameworks**		
	Mental Health Commission of Canada Toolkit	The toolkit can reduce clinician workload, ensure resources are evidence-based, increase interoperability, and provide valuable information for those with limited experience with digital mental health.
	Homewood Research Institute App Evaluation Framework	Used to evaluate the effectiveness of mental health apps and identifies the highest quality apps. Focuses on measuring outcomes, design and transparency, design methodology, and operational considerations of each application.
**Selection criteria**		
	Evidence base	Resources that are evidence-based and have supporting literature are often more accepted for use in clinical settings.
	Accessibility	Providers and researchers must consider the cost and technology requirements associated with recommending a digital intervention.
	Visual appeal	Mobile apps and web-based resources should host bright and catchy graphics, including music and characters, to grab the attention of youth.
	Feedback from youth	Recommendations should be developed based on feedback from youth about what they are looking for and the apps they are already using.
Cultural relevance		When serving youth, including BIPOC,^a^ further considerations must be made to ensure that the digital resource/intervention considers specific needs of various cultural and racial minority groups.

^a^BIPOC: Black, Indigenous, and people of color.

The discussions with stakeholders also identified new trends and innovations these individuals and organizations have seen in the youth digital mental health space. Table 2 highlights the most frequently cited innovations considered in response to the COVID-19 pandemic and other trends currently being studied in Canada.

**Table 2:** Upcoming trends and innovations in digital youth mental health identified through stakeholder interviews.

Trend or innovation	Description
Artificial intelligence/ machine learning	Including mobile apps that use artificial intelligence–powered chatbots.
Digital phenotyping	Tracking the activities of users to determine how digital activity might indicate a change in behavior or mental state.
Personalized therapy	Interventions that consider culture, language, geography, and more.
Interoperability	Using data from mobile apps alongside clinical data from health information systems.
Online peer support groups	Online groups moderated by health professionals to create safer digital environments for youth.
Trauma-informed interventions	Emerging mobile apps that target specific experiences with trauma.
Increasing accessibility	Potential to reach a greater number of children and youth through digital means alongside traditional in-person care.
Gamification	Development of video and digital games used to provide support and education about mental health and wellness.
Social media	The use of platforms such as Instagram and Twitter to help organizations reach youth and promote the availability of digital health interventions and resources. Instagram was also used in specific cases by youth organizations to provide mental health support through the application’s direct messaging feature.
TikTok	Includes TikTok accounts that provide short and accessible videos about mental health and wellness that can easily reach youth who are already using the app.

### Social Media Review

A total of 2184 tweets containing content or hashtags relating to digital interventions for youth mental health were included. Of these tweets, themes included promotion of regional chat services, such as text and telephone lines available 24 hours per day, 7 days per week for youth mental health support. Effects of isolation due to COVID-19 on youth mental health were highlighted, primarily concerning families who have experienced suicide and self-harm in youth [22]. A greater need for access to mental health care and reduction of hospital beds available for youth mental health due to staff shortages in New Brunswick was also a prominent topic [23]. Web-based resource hubs, webinars, and virtual panel discussions provided by youth mental health organizations were frequently promoted. Advocacy for government funding due to an increasing national need for youth mental health care was also discussed. Lastly, resources specifically designed to meet the needs of lesbian, gay, bisexual, transgender, queer, and two-spirit (LGBTQ2S+) youth were also promoted on the platform through youth-friendly infographics. 

## Discussion

### Future Directions

Although digital mental health interventions can provide great benefit during the COVID-19 pandemic, there is concern among some clinicians and researchers about the sustainability of these interventions once in-person care resumes across Canada. As digital mental health interventions are developed for youth, there needs to be increased awareness of how these interventions are being selected and the opportunities to better integrate these interventions with clinical care. One consideration includes improved transparency and quality considerations in how digital mental health interventions are selected, as shown in Table 1. Individuals involved in the development of digital resources for youth mental health must consider these selection criteria, such as cost, technology, privacy requirements, evidence base, visual appeal, and cultural relevance. Engagement with youth in planning and improving the integration of digital health with organizations across the mental health system continuum is vital to ensuring the longevity of these resources following the pandemic. Additionally, further research should be done to review content related to digital mental health on other social media platforms that are frequently used by youth, including Instagram and TikTok.

### Conclusions

Current literature, key stakeholder interviews, and mining of a social networking platform all point to the need for a greater understanding of digital intervention use for youth mental health during and beyond the COVID-19 pandemic. The use of digital interventions such as web-based resources and apps can be driven using suitable frameworks and selection criteria. Upcoming trends and innovations indicate an opportunity to improve and expand the use of digital interventions for youth in Canada.

## Appendix

Multimedia Appendix 1 Web-based resources identified for youth mental health in Canada, including criteria and tags.

Multimedia Appendix 2 Mobile apps identified for youth mental health in Canada, including criteria and tags.

## Abbreviations

LGBTQ2S+: lesbian, gay, bisexual, transgender, queer, and two-spirit
TAGS: Twitter Archiving Google Sheet
